# A Rolling-Bearing-Fault Diagnosis Method Based on a Dual Multi-Scale Mechanism Applicable to Noisy-Variable Operating Conditions

**DOI:** 10.3390/s25154649

**Published:** 2025-07-27

**Authors:** Jing Kang, Taiyong Wang, Ye Wei, Usman Haladu Garba, Ying Tian

**Affiliations:** School of Mechanical Engineering, Tianjin University, Tianjin 300354, China; tywang@tju.edu.cn (T.W.); yewei0116@tju.edu.cn (Y.W.); usmanhaladu88@tju.edu.cn (U.H.G.)

**Keywords:** fault diagnosis, VMD, CNN, rolling bearings, multi-scale signal denoising, multi-scale feature extraction and dynamic weighting

## Abstract

Rolling bearings serve as the most widely utilized general components in drive systems for rotating machinery, and they are susceptible to regular malfunctions. To address the performance degradation encountered by current convolutional neural network-based rolling-bearing-fault diagnosis methods due to significant noise interference and variable working conditions in industrial settings, we propose a rolling-bearing-fault diagnosis method based on dual multi-scale mechanism applicable to noisy-variable operating conditions. The suggested approach begins with the implementation of Variational Mode Decomposition (VMD) on the initial vibration signal. This is succeeded by a denoising process that utilizes the goodness-of-fit test based on the Anderson–Darling (AD) distance for enhanced accuracy. This approach targets the intrinsic mode functions (IMFs), which capture information across multiple scales, to obtain the most precise denoised signal possible. Subsequently, we introduce the Dynamic Weighted Multi-Scale Feature Convolutional Neural Network (DWMFCNN) model, which integrates two structures: multi-scale feature extraction and dynamic weighting of these features. Ultimately, the signal that has been denoised is utilized as input for the DWMFCNN model to recognize different kinds of rolling-bearing faults. Results from the experiments show that the suggested approach shows an improved denoising performance and a greater adaptability to changing working conditions.

## 1. Introduction

Rolling bearings play a pivotal role in the transmission systems of rotating machinery, acting as essential components that facilitate the interaction between stationary and rotating parts. Their widespread utilization in various applications underscores their importance in ensuring efficient performance and smooth operation within mechanical systems. By providing a supportive interface, rolling bearings not only enhance the functionality of machinery but also contribute to the longevity and reliability of the equipment in which they are incorporated. They are also essential operational components in large, high-end equipment, such as trains, processing machinery, wind turbines, and aircraft engines, where their failure directly impacts production safety, personnel safety, and operational efficiency [1,2,3]. Rolling bearings operate for extended periods in complex environments characterized by high noise levels, strong interference, and variable conditions. Consequently, the collected vibration signals are significantly affected by background noise and changing conditions, making the extraction of fault-sensitive features more challenging. Thus, eliminating noise interference from vibration signals and improving diagnostic accuracy under time-varying conditions are two pressing issues that need to be addressed [4,5].

At present, adaptive signal decomposition techniques have gained significant traction in the realm of fault diagnosis for rolling bearings [6], including Empirical Mode Decomposition (EMD) [7], Ensemble Empirical Mode Decomposition (EEMD) [8], Intrinsic Time-Scale Decomposition (ITD) [9], Variational Mode Decomposition (VMD) [10], etc. Variational Mode Decomposition (VMD) is an effective method for analyzing complex signals with nonlinear and non-stationary characteristics. Compared to other signal decomposition techniques, it offers advantages in noise resistance and enhanced feature separation, and has been successfully applied to the decomposition of fault signals in rolling bearings. VMD decomposition necessitates the construction and resolution of a constrained variational model, which is designed to yield multiple intrinsic mode function (IMF) components characterized by particular sparsity levels. This process requires careful consideration and expertise, as the determination of the appropriate number of decomposition levels must be made manually. It is crucial to recognize that selecting values that are either excessively small or excessively large can significantly influence the outcomes of the decomposition. Therefore, expert judgment plays a vital role in ensuring that the chosen parameters are optimal for achieving accurate and effective results in the VMD process. Consequently, much of the current literature on VMD focuses on determining and optimizing the number of decomposition modes. Lv et al. [11] introduced an innovative approach for fault feature extraction utilizing Adaptive Variational Mode Decomposition (AVMD). This method focuses on optimizing several critical parameters, including the number of modes *K*, the objective function known as Multi-Scale Fuzzy Entropy MFE, the embedding dimension *M*, the scale factor *S*, and the time delay *T*. By combining AVMD with the optimized Multi-Scale Fuzzy Entropy, their research enhances the precision of fault detection processes. In a related study, Li et al. [12] developed a cutting-edge weighted Variational Mode Decomposition (VMD) multi-point fusion technique aimed at improving the fault diagnosis accuracy specifically for spline tooth root crack fusion signals. This method demonstrates an advanced approach to analyzing complex signals, thereby contributing significantly to the field of fault diagnosis in mechanical systems. Addressing the challenges associated with insufficient decomposition and mode mixing in the VMD algorithm, Jin et al. [13] investigated the impact of improperly selecting the mode component *K* and the penalty factor α on diagnostic accuracy. They implemented the Whale Optimization Algorithm (WOA) to fine-tune these parameters, resulting in a marked improvement in the accuracy of rolling-bearing-fault diagnosis, thus showcasing the effectiveness of optimization algorithms in enhancing diagnostic techniques. Furthermore, Shi et al. [14] proposed an innovative approach that employs the Niche Genetic Algorithm (NGA) for optimizing the VMD method. This optimization was applied to extract faults from both simulated and real measurements of rolling-bearing signals, revealing the potential of the NGA to enhance the robustness of fault extraction methodologies. Lastly, Zhou et al. [15] utilized the Weighted Gray Wolf Optimization Algorithm (WGWOA) to optimize the parameters *K* and α within the VMD framework. Their experimental findings demonstrated that the WGWOA-optimized VMD successfully mitigates mode aliasing, thereby validating the efficacy of this optimization strategy in improving the performance of fault diagnosis processes. In summary, current research primarily focuses on resolving the optimization issues of decomposition parameters. Each type of mode component obtained through VMD decomposition contains signals of different scales. To achieve optimal denoising of the signal, it is crucial to eliminate noise from the signals corresponding to different scale mode components. Nevertheless, there is a paucity of discourse on denoising strategies leveraging the information embedded in these multi-scale mode components. In this paper, we propose a multi-scale denoising method that targets the denoising of mode components at different scales, yielding the most accurate denoised signal.

Compared to traditional shallow machine learning algorithms, neural network-based methods for bearing-fault diagnosis can adaptively extract deeper, more intrinsic features inherent in the data based on its structure and characteristics, facilitating fault classification based on these features [16,17]. In contrast to manual feature selection, which requires predefined fixed feature metrics, neural networks can adaptively extract optimal features that represent the signal characteristics, thereby minimizing the influence of human factors [18,19,20]. In the realm of research dedicated to the fault diagnosis of rolling bearings, particularly those functioning under substantial noise and fluctuating conditions, Sun et al. [21] introduced a novel approach known as the efficient multi-scale convolutional neural network (EMSCNN). This innovative model was specifically designed to combat the challenges associated with diagnosing faults in environments characterized by high levels of noise, which often result in diminished accuracy of detection. A significant aspect of the EMSCNN is its incorporation of interpretability visualization, which enhances the understanding of the model’s decision-making process and ultimately contributes to more reliable diagnostic outcomes. Additionally, Jin et al. [22] contributed to the field by developing a Multi-layer Adaptive Convolutional Neural Network (MACNN) model. This model was engineered to bolster diagnostic capabilities when faced with varying operational conditions, thereby improving the model’s overall adaptability to unexpected changes in the environment. By focusing on enhancing the responsiveness of diagnostic methods, the MACNN represents a noteworthy advancement in the ability to accurately assess the health of rolling bearings, even when the operating circumstances shift significantly. Furthermore, in their pursuit of advanced fault diagnosis techniques, Li et al. [23] proposed an enhanced multi-scale decomposition method that integrates convolutional neural networks. This sophisticated approach is aimed at effectively extracting fault features from multiscale infrared images, which are valuable for monitoring the health of gearboxes. By combining the strengths of multi-scale decomposition with the analytical power of convolutional neural networks, this method offers a promising avenue for improving gearbox health assessments, ensuring that potential issues are identified and addressed promptly. In a variable operating environment, the characteristics of bearing-fault signals in the time domain manifest as fault features appearing across different time scales, while in the frequency domain, these features emerge in various frequency bands [24]. When operating conditions change randomly and are accompanied by strong noise interference, it becomes difficult to determine which time scale or frequency band is more sensitive to fault characteristics. To comprehensively extract fault-sensitive features under varying conditions, multi-scale feature learning should be implemented [25,26,27]. Multi-scale features exhibit varying levels of sensitivity to different fault types under various operating conditions. To determine the sensitivity level of each fault type, dynamic weighting of the multi-scale features should be implemented, applying adaptive weights to these features [28,29,30,31,32]. In this paper, we propose a Dynamic Weighted Multi-Scale Feature Convolutional Neural Network (DWMFCNN) model, which is capable of extracting richer and more comprehensive multi-scale features. By integrating a mechanism that dynamically adjusts weight based on attention, the model increases its responsiveness to fault-related information while decreasing its sensitivity to fluctuations in operating conditions. This makes it appropriate for tasks involving the diagnosis and identification of bearing faults in varying environments.

To tackle the aforementioned challenges, this paper introduces a dual multi-scale mechanism for diagnosing faults in rolling bearings, designed to be effective in fault diagnosis tasks amidst noisy and fluctuating operating environments. This method integrates two innovations: multi-scale signal denoising and dynamic weighting of multi-scale feature extraction, focusing on solving the following two problems: (1) To address the issue of traditional VMD decomposition methods losing substantial genuine signal information by employing a local reconstruction strategy to reject noise modes, this paper proposes a method for multi-scale denoising of vibration signals based on AD distance fitting goodness-of-fit testing. (2) To tackle the problem of the significantly reduced fault diagnosis performance of neural network-based models under unknown operating conditions, this paper introduces a convolutional neural network structure that employs dynamic weighting of multi-scale features for the extraction and modulation of features from the denoised signals. The main contributions of this work are summarized as follows:

(1) A multi-scale denoising method based on AD distance fitting goodness-of-fit testing is proposed, where the original signal is decomposed using VMD, allowing for the removal of noise from mode components at multiple scales. This enables a closer estimation of the denoised reconstructed signal to the original signal, effectively separating noise from the mixed multi-scale mode components.

(2) A Dynamic Weighted Multi-Scale Feature Convolutional Neural Network (DWMFCNN) model is proposed, which learns rich and complementary fault-sensitive features in parallel across multiple time scales and applies dynamic weighting. This improves the sensitivity to fault characteristics while diminishing features that are sensitive to conditions, consequently enhancing the model’s ability to adapt across different domains and increasing the accuracy of fault diagnosis under diverse circumstances.

(3) The proposed dual multi-scale mechanism for bearing-fault diagnosis is evaluated using the CWRU dataset and comprehensive simulated mechanical fault tests from Spectra Quest (SQ). Experimental results indicate that the method effectively achieves multi-scale denoising, feature extraction, and dynamic weighting, making it suitable for bearing operating environments characterized by high noise interference and variable conditions.

## 2. Methods and Theory

### 2.1. Anderson–Darling (AD)

The AD test statistic is a statistical distance metric that estimates the degree to which an observed dataset follows a given target function distribution [33]. This method measures the integral of the squared distance between the empirical cumulative distribution function (EDF) and the target cumulative distribution function (CDF) to estimate the fit between the given target CDF and the current observed data EDF [34]. Assuming a given target CDF G(x) and observed data EDF E(x), the AD test statistic is defined as follows:

(1) $$\Delta ={\int |G\left(x\right)-E\left(x\right)|}^{2}W\left(x\right)dG\left(x\right)=\int \frac{{\left[G\left(x\right)-E\left(x\right)\right]}^{2}}{E(x)(1-E(x))}dG\left(x\right)$$

Here, E(x) represents the empirical cumulative distribution function of the observed data, G(x) denotes the target cumulative distribution function, and W(x) is the weight function used to restrict the location of the data. Additionally, $W\left(x\right)=\frac{1}{E(x)(1-E(x))}$. Due to the practical difficulty of computing the true theoretical representation in Equation (1), Anderson and Darling proposed a computable numerical formula in 1954 as follows [35]:

(2) $$\Delta =-n-\frac{1}{n}\sum_{t=1}^{N}\left(2t-1\right)[\log G\left(x\left(t\right)\right)+\log\left(1-G\left(x\left(N-t+1\right)\right)\right]$$

Here, x(t) represents a set of observed data of length *N*. A small AD statistic indicates that the data conforms more closely to the distribution. The AD test uses threshold comparison with parameter σ to assess data fit to the target function. A smaller σ value is chosen to enhance noise component detection in the data.

### 2.2. Variational Mode Decomposition (VMD)

Variational Mode Decomposition (VMD) is an advanced signal decomposition technique designed to systematically analyze and decompose an input signal into *K* distinct components, each exhibiting sparse properties. This innovative method operates through an iterative search process aimed at identifying the optimal solution within a variational framework. This process is crucial, as it facilitates the accurate determination of both the center frequency and bandwidth associated with each individual component of the signal. As a result, VMD not only allows for the effective separation of intrinsic mode functions (IMFs) but also enables the frequency domain partitioning of the overall signal. Consequently, this technique provides a robust and efficient means of decomposing the given signal [36]. Assume that the input signal s(t) consists of *K* mode components $\nu_{k}\left(t\right)$ with finite bandwidth, where each mode component has a center frequency of ω(t). The constraint is that the sum of the modes τ(t) equals the input signal. The specific decomposition steps are as follows:

(1) The analytical signal $\nu_{k}\left(t\right)$ is obtained through Hilbert transform, and its one-sided spectrum and the square norm $L^{2}$ of the demodulated gradient are computed to estimate the bandwidth of each mode component:

(3) $$\left\{\begin{array}{cc} & \min_{\left\{v_{k}\right\}\left\{\omega_{k}\right\}}\left\{\sum_{k=1}^{K}{∥\partial t\left[\left(\delta \left(t\right)+\frac{j}{\pi t}\right)*v_{k}\left(t\right)\right]e^{-j\omega_{k}t}∥}^{2}\right\}\\ & s.t.\sum_{k=1}^{K}v_{k}\left(t\right)=f\left(t\right) \end{array}\right.$$

In the equation, $\left\{\nu_{k}\right\}=\left\{\nu_{1},\ldots ,\nu_{k}\right\}$ represents the decomposed intrinsic mode function (IMF) component, while $\left\{\omega_{k}\right\}=\left\{\omega_{1},\ldots ,\omega_{k}\right\}$ denotes the center frequency of each IMF component.

(2) By introducing the Lagrange multiplier τ(t) and a second-order penalty factor α, the constrained variational problem is transformed into an unconstrained one. The Lagrange multiplier assures the rigidity of the constraint conditions, whereas the second-order penalty factor secures precise signal reconstruction within a Gaussian noise setting. The expression for the extended Lagrangian is presented as follows:

(4) $$\begin{array}{cc} & L\left(\left\{\nu_{\dot{k}}\right\},\left\{\omega_{\dot{k}}\right\},\tau \right)=\alpha \sum_{\dot{k}}{∥\partial_{t}\left[\left(\delta \left(t\right)+\frac{j}{\pi t}\right)*\nu_{\dot{k}}\left(t\right)\right]e^{-jw_{\dot{k}}t}∥}^{2}\\ & +{∥s\left(t\right)-\sum_{\dot{k}}\nu_{\dot{k}}\left(t\right)∥}^{2}+\langle \tau \left(t\right),s\left(t\right)-\sum_{\dot{k}}\nu_{\dot{k}}\left(t\right)\rangle \end{array}$$

(3) The Alternating Direction Method of Multipliers (ADMM) serves as an effective technique for the continual updating of each mode component, along with its corresponding center frequency. This iterative process ultimately leads to the identification of the saddle point of the unconstrained model, which signifies the optimal solution to the problem at hand. The detailed procedure for this updating process is outlined as follows:

(5) $$\begin{array}{cc} \; & {\hat{v}}_{k}^{n+1}\left(\omega \right)=\frac{\hat{s}\left(\omega \right)-\sum_{i\neq k}{\hat{v}}_{i}\left(\omega \right)+\hat{\tau }\left(\omega \right)/2}{1+2\alpha {\left(\omega -\omega_{k}\right)}^{2}}\\ \; & \omega_{k}^{n+1}=\frac{\int_{0}^{\infty }\omega |\nu_{k}^{n+1}\left(\omega \right){|}^{2}d\omega }{\int_{0}^{\infty }|\nu_{k}^{n+1}\left(\omega \right){|}^{2}d\omega }\\ \; & {\hat{\tau }}^{n+1}\left(\omega \right)={\hat{\tau }}^{n}\left(\omega \right)+\tau \left(\hat{s}\left(\omega \right)-\sum_{k}{\hat{\nu }}_{k}^{n+1}\left(\omega \right)\right) \end{array}$$

Repeat Equations (4) and (5) until the stopping criteria for the iterative updates are satisfied:

(6) $$\sum_{k}∥{\hat{\nu }}_{k}^{n+1}-{\hat{\nu }}_{k}^{n}{∥}_{2}^{2}/{∥{\hat{\nu }}_{k}^{n}∥}_{2}^{2}<\varepsilon$$

In Equations (4) and (5), ω represents the frequency, while $\nu_{k}^{n+1}\left(\omega \right)$, $\hat{s}\left(\omega \right)$ and $\hat{\tau }\left(\omega \right)$ are the Fourier transforms corresponding to $\nu_{k}^{n}\left(t\right)$, s(t), and τ(t).

### 2.3. Convolutional Neural Network

A convolutional neural network (CNN) is a sophisticated type of deep neural network characterized by its convolutional architecture. This structure is particularly effective in identifying and utilizing local features within data, which facilitates the extraction of overarching training features and enhances classification performance. One of the distinguishing attributes of a CNN is its weight-sharing mechanism, which evokes similarities with biological neural networks. This unique design enables CNNs to perform exceptionally well in various applications, particularly in the domains of data classification and pattern recognition. The architecture of a CNN is fundamentally organized into two main stages: feature extraction and classification. In practice, this procedural flow encompasses several critical operations, including convolution, pooling, and activation functions, followed by one or more fully connected layers. Each of these steps plays a vital role in refining the input data and enabling the network to make accurate predictions. The mathematical representation of this framework provides a clear understanding of how each component interrelates to contribute to the overall effectiveness of the network [37].

(1) Convolutional Layer:

(7) $$z_{k}^{l}=a_{k}^{l-1}*w_{k}^{l}+b_{k}^{l}$$

where $z_{k}^{l}$ denotes the *k*-th feature map of the *l*-th convolutional layer; $w_{k}^{l}$ and $b_{k}^{l}$ are the weights and biases of the *k*-th feature map in the *l*-th convolutional layer, respectely; and * denotes the convolution operation.

(2) Pooling Layer:

(8) $$y_{i,j,k}^{l}={\mathrm{downsample}}_{\left(m,n\right)\in R_{i,j}^{l}}^{l}\left(x_{m,n,k}^{l}\right)$$

where downsample(·) represents the down-sampling rule, $y_{i,j,k}^{l}$ is the new value at location (i,j) in the *k*-th feature map of the *l*-th layer after the pooling operation, $R_{i,j}^{l}$ is the pooling receptive region around location (i,j) and $x_{m,n,k}^{l}$ is the node at location (m,n) within the receptive field.

(3) Activation Layer:

(9) $$a_{k}^{l}=\sigma \left(z_{k}^{l}\right)$$

where $a_{k}^{l}$ is a nonlinear feature value of the *k*-th feature map, and σ(·) is the activation function expressed as a rectified linear unit.

(4) Fully Connected Layer:

(10) $$full_{k}^{l}=\sigma \left(a_{k}^{l-1}\times w_{k}^{l}+b_{k}^{l}\right)$$

where *y* represents the predicted labels, $w_{k}^{l}$ is the weight matrix of the fully connected layer, and $b_{k}^{l}$ denotes the bias.

## 3. The Proposed Diagnosis Method

### 3.1. Multi-Scale Denoising Method

The advantage of VMD lies in its ability to separate noise from the true signal. Based on the AD statistical distance curve, the Intrinsic Mode Functions (IMFs) decomposed through VMD can be categorized into three groups: (1) pure real signal modes; (2) modes that include both noise and real signals; and (3) pure noise modes [38].

**Figure 1:**
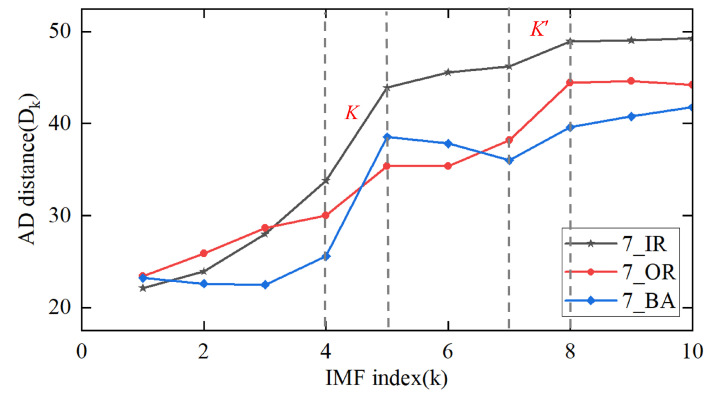
AD statistical distance change trend curve.

The correlation signal pattern is determined by assessing the gradient of the distance between consecutive Intrinsic Mode Functions (IMFs). A pronounced alteration in gradient between adjacent IMFs indicates a sudden decrease in signal information from earlier to later IMFs, suggesting a further decline in signal fidelity with escalating noise in subsequent IMFs. Current approaches utilize the maximal gradient, denoted as *K*, in the distance profile to identify pure, real signal modes [39,40]. Consequently, IMFs are segregated into distinct categories of pure, real signal modes and noisy modes based on the gradient *K*. Within the noisy modes, a peak slope $K^{\prime }$ is identified, representing the point of maximum transition between the partially noisy mode and the pure noisy mode. In other words, the pure noise mode is distinguished from modes containing a mixture of signal and noise by the slope $K^{\prime }$ within the noisy modes. For example, by analysing different fault type signals 7-OR, 7-IR, and 7-BA, we derived the trend of statistical distance changes after each IMF fault, as shown in Figure 1.

Figure 1 shows that the largest slope in the second (mixed signal and noise mode) and third (pure noise mode) classes of each signal occurs between the seventh and eighth IMFs. This shows that the signal content is concentrated in the first eight IMFs, followed by a pure noise pattern.

**Figure 2:**
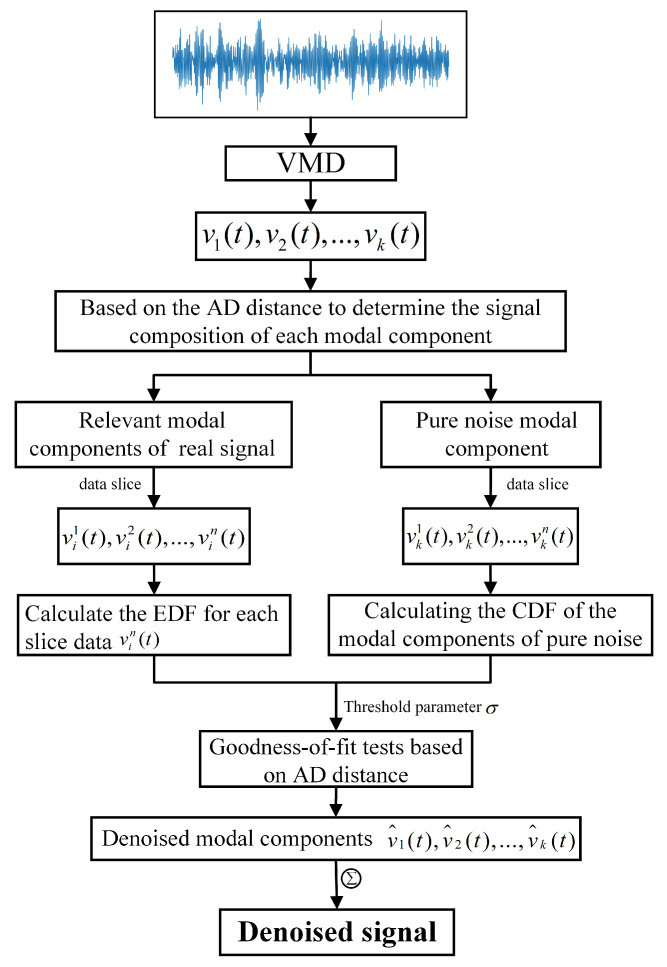
Multi-scale signal-denoising method based on AD test.

Traditional VMD decomposition reconstructs the denoised signal by rejecting modes of the second and third types and accepting only the first type (pure true signal mode). However, the second type also contains a significant amount of true signal content, and rejecting it can lead to a substantial loss of valuable information. To tackle this problem, this section introduces a multi-scale denoising approach that utilizes the AD-distance-fitting goodness-of-fit test (AD test), as demonstrated in Figure 2.

The multi-scale denoising method based on AD test includes the following specific steps:

(1) For the second and third types of intrinsic mode functions, the AD statistic is used to calculate the statistical distance S(i) between the first *i* modes (where i≤K−1 ) and the *K*-th mode $\nu_{k}\left(t\right)$. This results in the AD distance trend curve for each mode. The calculation formula for S(i) is as follows:

(11) $$\begin{array}{cc} \; & S_{i}=-n-\frac{1}{n}\sum_{t=1}^{n}\left(2t-1\right)\left[\log E(\nu_{i}\left(t\right)+\log(1-E\left(\nu_{i}\left(n-t+1\right)\right]\right]\\ \; & E\left(z\right)=\frac{1}{n}\sum_{t=1}^{n}\left(\nu_{k}\left(t\right)\leq z\right) \end{array}$$

(2) For the second and third types of intrinsic mode functions obtained from VMD decomposition, the slope of their trend curves is used to identify other purely noise components: The slope of the distance between the *i*-th IMF and the (i+1)-th IMF is denoted as

(12) $$K_{i}=\left|S_{i+1}-S_{i}\right|$$

The maximum slope is denoted as $k^{\prime }=\max\left(K_{i},K_{i+1},\ldots K_{k}\right)$. Based on the maximum slope, the criteria for selecting the intrinsic mode functions related to the true signal and rejecting the purely noise mode components are established as follows:

(13) $$\left\{\begin{array}{c} \nu_{i}\left(t\right),i\leq K^{\prime }\in \mathrm{real}\;\mathrm{signal}\;\mathrm{correlation}\;\mathrm{mode}\;\mathrm{component}\\ \nu_{i}\left(t\right),i>K^{\prime }\in \mathrm{purely}\;\mathrm{noisy}\;\mathrm{modal}\;\mathrm{component} \end{array}\right.$$

(3) Estimate the noise distribution in the signal based on the rejected purely noise mode components. First, segment the pure noise mode components into data slices, then use the overall average of each slice’s empirical distribution function (EDF) to estimate the cumulative distribution function (CDF) of the overall pure noise mode components. The specific steps are as follows:

For each purely noise mode component $\nu_{i}\left(t\right)\left\{i>k^{\prime },t=1,\ldots ,N\right\}$, divide $\nu_{i}\left(t\right)$ into *M* data slices of a specified length *L*, represented as follows:

(14) $$\nu_{i}\left(t\right)=\left\{\begin{array}{c} \left\{v_{i}^{1}\left(t\right),t=1,...,L+1\right\},\\ \left\{v_{i}^{2}\left(t\right),t=L+1,...,2\left(L+1\right)\right\},\\ ...\\ \left\{v_{i}^{n}\left(t\right),t=\left(n-1\right)\left(L+1\right),...,n\left(L+1\right)\right\} \end{array}\right.$$

The EDF of the *n*-th data slice $\nu_{i}^{n}\left(t\right)$ is denoted as $E_{i}^{n}\left(z\right)$, and the calculation formula is as follows:

(15) $$E_{i}^{n}\left(z\right)=\frac{1}{L}\sum_{t=1}^{L+1}\left(\nu_{i}\left(t\right)\leq z\right),i>k^{\prime }$$

The approximate estimate of the cumulative distribution function (CDF) for the overall pure noise mode component is denoted as G(z), and the calculation formula is

(16) $$G\left(z\right)=\frac{1}{M(k-k^{\prime })}\sum_{i=k^{\prime }+1}^{k}\sum_{n=1}^{M}E_{i}^{n}\left(z\right)$$

(4) Use the AD test to detect noise in the mode components related to the real signal. First, segment each mode component related to the real signal into data slices, then calculate the AD statistical distance between the EDF of each slice and the CDF of the pure noise mode component obtained in step (3). This distance is compared with the threshold coefficient, and based on the comparison results, the degree of fit between the slice data and the pure noise distribution is determined. For each mode component related to the real signal $\nu_{i}\left(t\right)\left\{i\leq k^{\prime },t=1,\ldots ,N\right\}$, the calculation of the EDF for each slice is the same as in Equation (17); the AD statistical distance $\Delta_{i}^{n}$ between the EDF of each slice and the CDF of the pure noise mode component from step (3) is derived from Equation (2).

Set the threshold parameter $\sigma =10^{-4}$, which basically represents the maximum distance required for a close fit between the two EDFs. The denoised mode component ${\hat{\nu }}_{i}\left(t\right)$ can be evaluated for goodness of fit based on the distance $\Delta_{i}^{n}$.

(17) $$\left\{\begin{array}{c} \Delta_{i}^{n}\leq \sigma ,\mathrm{Closely}\;\mathrm{fitted}\;\mathrm{to}\;\mathrm{pure}\;\mathrm{noise}\;\mathrm{distributiom}\Rightarrow reject\\ \Delta_{i}^{n}>\sigma ,\mathrm{Not}\;\mathrm{fitted}\;\mathrm{to}\;\mathrm{pure}\;\mathrm{noise}\;\mathrm{distribution}\Rightarrow accept \end{array}\right.$$

(5) The representation of the denoised mode component ${\hat{\nu }}_{i}\left(t\right)$ obtained from step (4) is as follows:

(18) $$\left\{\begin{array}{c} \;\Delta_{i}^{n}\leq \sigma ,{\hat{\nu }}_{i}\left(t\right)=0\\ \Delta_{i}^{n}>\sigma ,{\hat{\nu }}_{i}\left(t\right)=\nu_{i}\left(t\right) \end{array}\right.$$

Finally, the signal is reconstructed using the denoised mode component ${\hat{\nu }}_{i}\left(t\right)$. The representation of the real signal $\hat{x}\left(t\right)$ obtained after applying the proposed denoising method to the signal x(t) is as follows:

(19) $$\hat{x}\left(t\right)=\sum_{k=1}^{k^{\prime }}{\hat{v}}_{i}\left(t\right),k\leq k^{\prime }$$

### 3.2. Multi-Scale Feature Extraction and Dynamic Weighting Method

**Figure 3:**
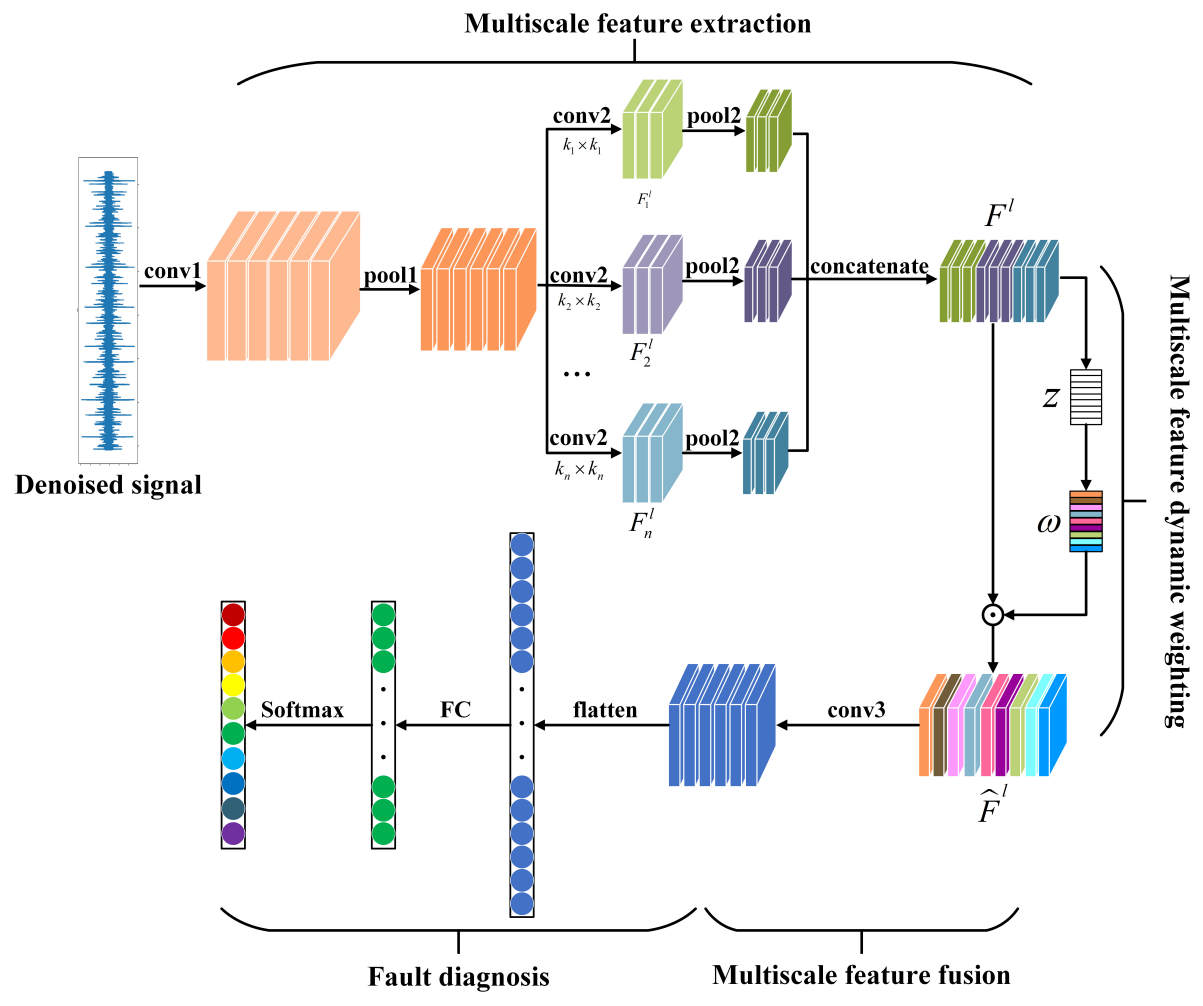
Network architecture of DWMFCNN.

In order to tackle the traits of bearing vibration signals across different operating conditions, this section introduces a Dynamic Weighted Multi-Scale Feature Convolutional Neural Network (DWMFCNN) model, specifically crafted for diagnosing and identifying bearing faults in fluctuating environments.The structure of the DWMFCNN model is illustrated in Figure 3, with the denoised true signal $\hat{x}\left(t\right)$ as the input and the fault type as the output. The DWMFCNN model consists of four components: multi-scale feature extraction module, multi-scale feature dynamic weighting module, multi-scale feature fusion module, and fault classification module.

(1) Multi-scale feature extraction module

The multi-scale feature extraction module begins by incorporating a convolutional layer and a pooling layer to transform the raw input vibration signals into shorter features, thereby accelerating the computation speed of subsequent network layers. It then establishes a multi-scale feature extraction layer to capture features at various scales. Recognizing that the characteristics of vibration data vary in fault sensitivity across different scales under various operating conditions, a multi-scale feature extraction layer is designed to capture fault features at different scales corresponding to varying rotational speeds and load conditions. Convolutional kernels of different widths provide distinct local receptive fields, allowing for the learning of features at multiple scales [24]. The multi-scale feature extraction layer consists of *n* parallel multi-scale feature extraction convolutional layers, each containing channels. Each parallel convolutional layer employs convolutional kernels of varying widths, denoted as $k_{i}\left\{i=1,2,\ldots ,n\right\}$, which are used to extract features at different scales. The features extracted by the *i*-th convolutional kernel are represented as follows:

(20) $$F_{i}^{l}=f\left(K_{i}*X^{l-1}+b_{i}\right)$$

Here, $F_{i}^{l}$ denotes the data features extracted by the *i*-th convolutional kernel in the *l*-th convolutional layer, $K_{i}$ represents the size of the *i*-th kernel, $X^{l-1}$ indicates the feature outputs from the previous layer, and $b_{i}$ refers to the bias term. The activation function *f* is commonly chosen as the ReLU activation function. The multi-scale features output from the *l*-th layer, denoted as $F^{l}$, can be expressed as follows:

(21) $$F^{l}=\left[F_{1}^{l},F_{2}^{l},\ldots ,F_{n}^{l}\right]$$

(2) Multi-scale feature dynamic weighting module

Following the multi-scale feature extraction layer, the attention mechanism is introduced to dynamically weight and modulate the extracted multi-scale data features. This results in a dynamically adjustable weight vector ω that controls the amount of information passed forward from each scale feature. The optimized multi-scale features enhance sensitivity to fault information while reducing sensitivity to varying operating conditions. According to the principle of SENet’s attention mechanism, $F^{l}$ is first compressed into a c×l feature vector *z* by a global average pooling operation, followed by a gate mechanism consisting of two layers of full connectivity, where the activation function of the first full connectivity layer is ReLU, and the activation function of the second full connectivity layer is Sigmoid. Finally, attention weights are applied. The weight vector ω is computed as follows:

(22) $$\begin{array}{cc} \; & z=F_{sq}\left(F^{l}\right)=\frac{1}{c\times l}\sum_{i=1}^{c}\sum_{j=1}^{l}F^{l}\left(i,j\right)\\ \; & \omega =\sigma (w_{2}\delta \left(w_{1}z\right)) \end{array}$$

where σ is the Sigmoid activation function, and δ is the ReLU activation function; $w_{1}$ and $w_{2}$ are the parameters of the two fully-connected layers, respectively; and the weight vector is ω $\in R^{C\times 1}$, where the *i*-th element represents the fault sensitivity of the multiscale feature in the *i*-th channel. The optimized multi-scale features ${\hat{F}}^{l}$, adjusted dynamically using the weight vector ω, are expressed as follows:

(23) $${\hat{F}}^{l}=F^{l}\cdot \omega$$

where ‘·’ denotes element-wise multiplication across channels.

(3) Multi-scale feature fusion module

To effectively capture the dependencies that exist among features across various scales and channels, a convolutional layer has been incorporated into the architecture. This addition plays a crucial role in enhancing the extraction process, allowing for the derivation of multi-scale fused features from the previously optimized multi-scale features.

(4) Fault classification module

The features resulting from multi-scale fusion are input into the fully connected layer and the Softmax layer, resulting in the output of fault types.

### 3.3. Rolling-Bearing-Fault Diagnosis Method Based on Dual Multi-Scale Mechanism Applicable to Noisy-Variable Operating Conditions

**Figure 4:**
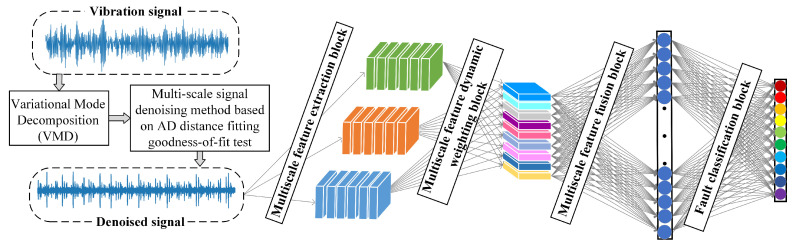
Framework of the proposed method.

Figure 4 illustrates the rolling-bearing-fault diagnosis method proposed in this paper based on the dual multi-scale mechanism applicable to noisy-variable operating conditions, which consists of two parts. The first part uses the signal multi-scale denoising method based on the AD-distance-fitting goodness-of-fit test to denoise the original vibration signals on a mode component scale to obtain the denoised signals. The second part proposes the DWMFCNN model, which takes the denoised signal as the input learning and discriminative features, integrates the two structures of multi-scale feature extraction and multi-scale feature dynamic weighting, which prompts the model to extract the fault-sensitive features that are not affected by the working environment, and improves the model’s performance in the diagnosis of faults against the changes in working conditions. In summary, the rolling-bearing-fault diagnosis method based on dual multi-scale mechanism proposed in this paper can not only achieve multi-scale denoising but also multi-scale feature extraction and dynamic weighting, and it is applicable to the service environment of rolling bearings with high noise and unstable working conditions.

## 4. Experimental Validation and Analysis

### 4.1. Description of Experimental Data

**Figure 5:**
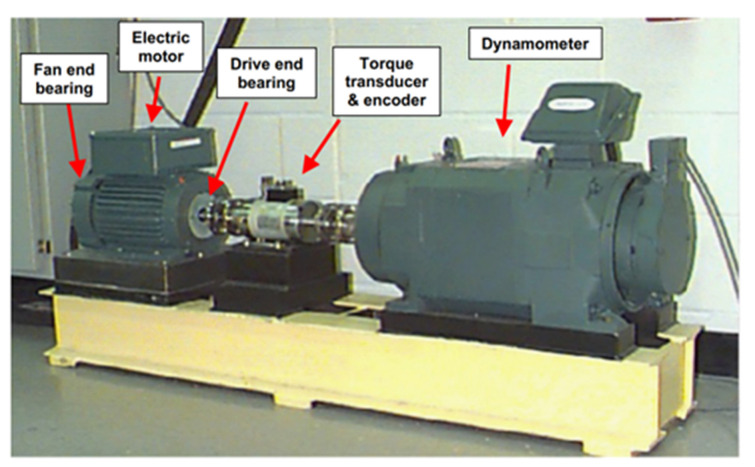
Experimental platform of CWRU bearing test [42].

In this paper, data from the Case Western Reserve University (CWRU) Bearing Data [41] Centre are used to experimentally validate and analyse the effectiveness of the bearing-fault diagnosis method based on the dual multiscale mechanism for fault diagnosis under noisy-variable operating conditions. The basic layout of the experimental test setup is shown in Figure 5. During the experiment, faults with diameters measuring 0.007, 0.014, 0.021, and 0.028 inches were introduced using electric discharge machining (EDM) in both the drive-end bearing (SKF deep groove ball bearing: 6205-2RSJEM) and the fan-end bearing (6203-2RSJEM) of the motor. These faults were positioned on the rolling element, the inner ring, and the outer ring, respectively. A comprehensive overview of the data set can be found in Table 1. Containing data from the normal condition of the bearing, the dataset can be categorized into ten fault types.

### 4.2. Denoising Effect of the Proposed Method in Noisy Environments

This section randomly selects two of the ten fault types from the Case Western Reserve University (CWRU) bearing dataset as samples, extracting 2048 data points from each to validate the denoising effectiveness of the proposed multi-scale denoising method in noisy environments. The selection of fault types is randomized to ensure the validation method’s generalizability. In practical industrial applications, environmental noise is inevitable. To simulate the effects of various random processes found in nature, Gaussian white noise is introduced to the raw vibration data of the chosen samples in this subsection. The signal-to-noise ratio (SNR) is defined as follows:

(24) $$SNR_{dB}=10\log_{10}\left(\frac{P_{signal}}{P_{noise}}\right)$$

where $P_{signal}$ and $P_{noise}$ denote the power of the original vibration signal and the additional Gaussian white noise, respectively.

The two randomly selected fault types are 7-BA and 21-OR, which are set as 9 and 7 mode components in the procedure for VMD decomposition [13], and the parameters for VMD decomposition of other fault types are also set in accordance with the parameters in the literature [13], which are all theoretically verified. The appropriate hyperparameters are chosen according to the relevant literature to obtain better performance and meet the experimental requirements.

**Figure 6:**
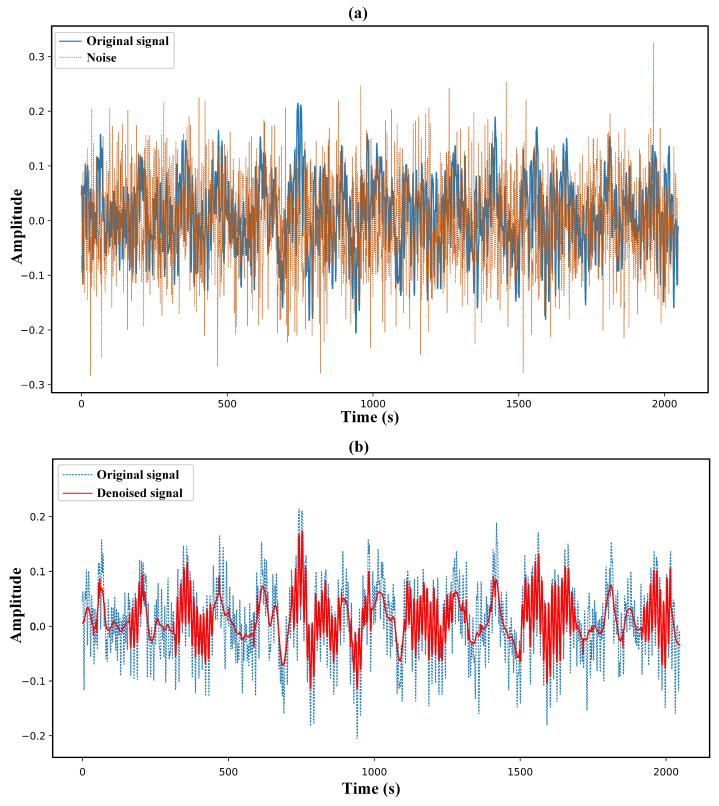
Multiscale denoising results of 7-BA type fault vibration signals. Subfigure (**a**) shows the added noise with SNR = 2 (dashed line) and its original vibration signal (solid line), while subfigure (**b**) shows the denoised signal by the proposed multiscale denoising method (solid line) and the original vibration signal as a reference (dashed line).

**Figure 7:**
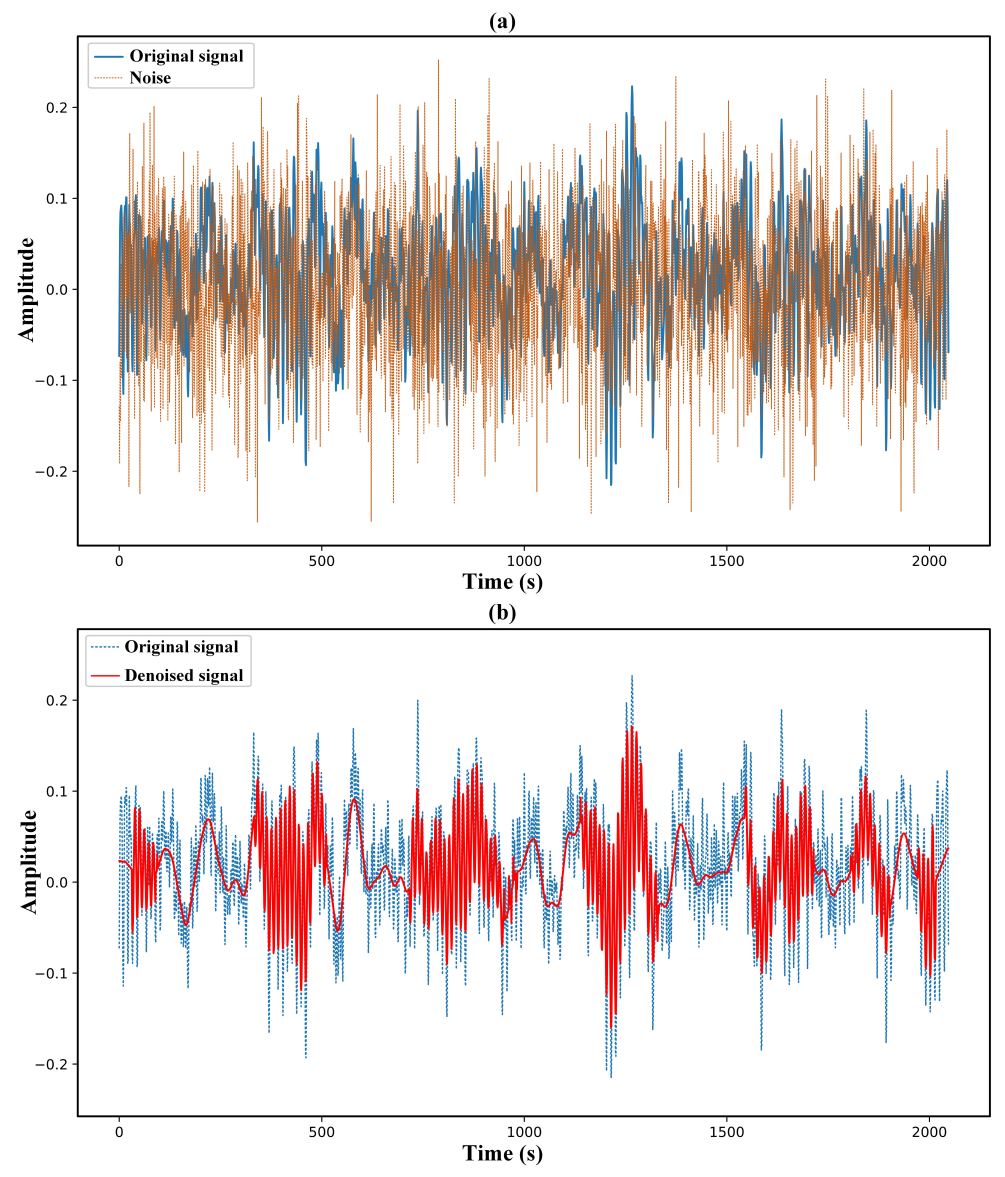
Multiscale denoising results of 21-IR type fault vibration signals. Subfigure (**a**) shows the added noise with SNR = −2 (dashed line) and its original vibration signal (solid line), while subfigure (**b**) shows the denoised signal by the proposed multiscale denoising method (solid line) and the original vibration signal as a reference (dashed line).

Figure 6 and Figure 7 present the signal curves resulting from the proposed multiscale denoising method applied to the bearing-vibration signals of the 7-BA and 21-IR fault types, respectively. The denoising effects observed in these figures demonstrate that the proposed multi-scale denoising method effectively performs well in real noisy environments. The denoised signals for the 7-BA and 21-IR fault types are shown in Figure 6b and Figure 7b, respectively, illustrating a close resemblance to their original signals. These results also capture important details, such as higher peaks and trends in the original signals, owing to the method’s capability to filter out coefficients resembling noise statistics at multiple scales (i.e., variousmode components). This process allows for the removal of noise-related elements and the reconstruction of denoised signals. The results affirm that the proposed method successfully retains the subtle variations of real signal components obscured by noise while effectively suppressing industrial environmental noise. Overall, the denoising outcomes for both fault types validate the method’s efficacy in maintaining the integrity of signal features in noisy conditions.

**Figure 8:**
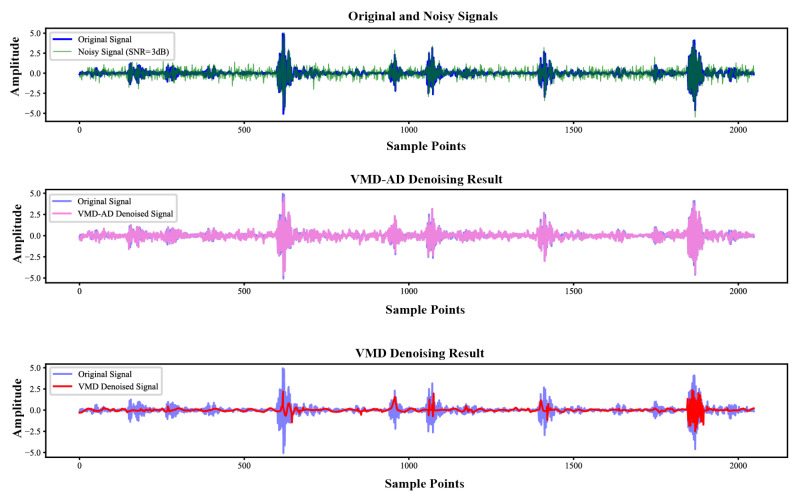
Comparison of denoising methods.

Figure 8 illustrates the impact of VMD-AD denoising on the 14-BA fault signal, while Table 2 presents the evaluation metrics for denoising methods. Analyses from Figure 8 and Table 2 reveal that the VMD-AD method exhibits a higher signal-to-noise ratio (SNR) improvement compared to the standard VMD approach, signifying enhanced signal quality. Moreover, the VMD-AD method yields a lower root mean square error (RMSE), indicating higher denoising accuracy and reduced distortion in the signal.

### 4.3. The Effectiveness of the Proposed Method in Fault Diagnosis Under Varying Operating Conditions

#### 4.3.1. The Effectiveness of the Proposed Method for Fault Diagnosis Under Variable Load Conditions

This section uses fault vibration data from the drive-end bearing measured in the CWRU bearing dataset to demonstrate the effectiveness of the proposed method for fault classification tasks under variable working conditions, including fault data type description, fault diagnosis task settings, and comparative analyses. The test-sampling frequency is 12 kHz, and 300 samples are selected for each fault state and motor load condition, with 2048 data points collected for each sample. This section presents the fault vibration data from the drive end bearings, measured using the CWRU bearing dataset, to validate the effectiveness of the proposed methodology for fault classification in a variable operating condition environment. It includes descriptions of the fault data type, the setup for the fault diagnosis task, and a comparative discussion. The sampling frequency for the tests was set at 12 kHz. To simulate different operating conditions, each faulty bearing was mounted identically on the test bench and operated at a constant speed under motor loads ranging from 0 to 3 hp. In this experiment, 300 samples were selected for each fault condition across all motor load conditions, with 2048 data points extracted for each sample. A detailed description of the data is provided in Table 1, and the data aligns with the specific working conditions and fault diagnosis tasks outlined in Table 3. Task T1 indicates that the proposed model is trained on sample data under 0 hp, 2 hp, and 3 hp loads and is subsequently tested with sample data at a 1 hp load. Similarly, Task T2 and Task T3 are trained with sample data under three different loads, and then the model is tested with data different from these three loads. Each task includes 9000 samples for training and 3000 samples for testing. The ratio of training to test sets is 3:1.

The settings of each parameter of the proposed DWMFCNN model are shown in Table 4. In order to verify the effectiveness of the model in the fault classification task under variable load operating conditions environment, two models, the MC-CNN model [25] and the CNN model, are selected for comparison experiments: The MC-CNN and CNN models have the same model depth as the proposed DWMFCNN model; the MC-CNN model adopts the structure given in the literature, and the CNN model consists of three pairs of Conv2d layer and pooling layers. The convolutional kernel is the same size as the second convolutional layer in the proposed model, and the pooling layer is the same size as the second pooling in the proposed model. Similarly, the comparison models also use the same training samples as input for the fault diagnosis task. All the models in this section of experiments are built under the Keras framework, the batch size of the models is set to 128, the epoch is set to 20, and the optimization algorithm is Adam.

**Figure 9:**
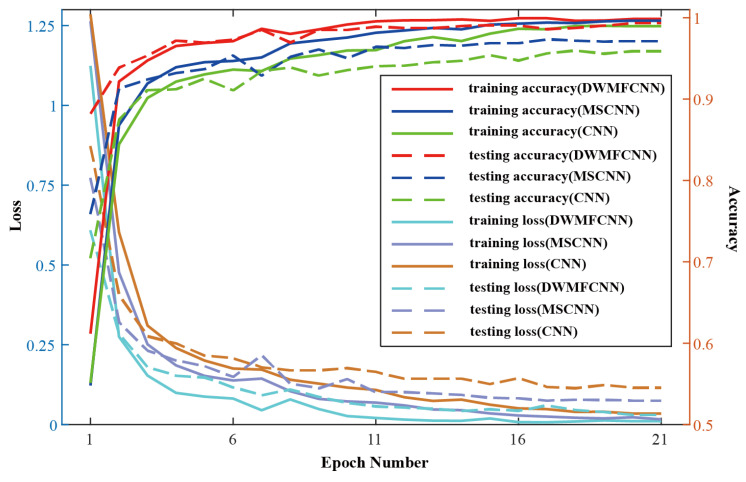
Model convergence performance curves of CNN, MC-CNN and DWMFCNN in Task T1.

Figure 9 shows the model convergence performance curves of the proposed DWMFCNN model and the two comparison models in task T1, which are obtained by averaging the results of 10 repetitive experiments to mitigate the effect of model stochastic performance. From the test results of the three models, it can be seen that the proposed DWMFCNN model outperforms the other two models, with a fault diagnosis accuracy of 99.68% in task T1. From the four curves of training accuracy, testing accuracy, training loss, and testing loss of DWMFCNN, it can be seen that the model can achieve the highest accuracy and the lowest loss among the three models in a small epoch, which verifies the significance of the dynamic weighted modulation of multi-scale features in the model and improves the ability of the model to learn multi-scale features to classify the ten bearing-fault types. Also, from the training and testing losses of the three models, it can be seen that CNN, MC-CNN, and DWMFCNN do not overfit, and after a series of epochs, the testing losses of each model converge to a certain level, so the accuracy of each model is reliable for the performance comparison.

**Figure 10:**
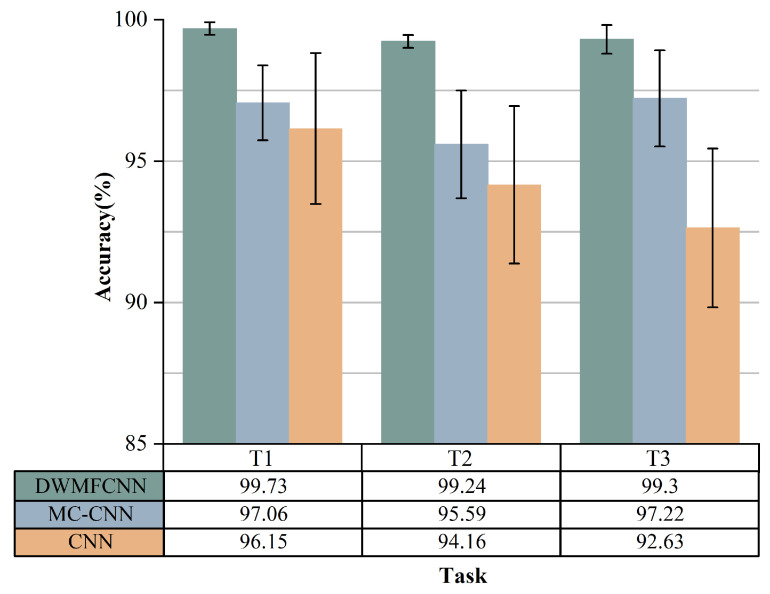
Performance of the proposed DWMFCNN model in three tasks (T1–T3) compared to CNN and MC-CNN.

Figure 10 illustrates that the proposed DWMFCNN model achieves the highest fault diagnosis accuracy and the lowest standard deviation across tasks T1, T2, and T3, with an accuracy exceeding 99% in all three tasks. The following comparative insights can be drawn: (1) the model demonstrates superior adaptability to variable load conditions compared to the other two models; (2) it exhibits a higher fault diagnosis accuracy relative to the other models; (3) the DWMFCNN model shows greater stability than its counterparts. Among the three models, the CNN model performs the least effectively due to the domain drift issue [43] in fault diagnosis tasks under varying load conditions, which diminishes the trained CNN’s diagnostic capability in new scenarios and limits its ability to adjust to load changes. In contrast, the MC-CNN model outperforms the CNN model in fault diagnosis accuracy, as it can extract a more diverse set of faults across multiple scales, allowing multi-scale features to better represent fault characteristics under varying load conditions compared to single-scale features. Although MC-CNN and CNN achieve high accuracy in Task T1, their performance drops in Tasks T2 and T3, highlighting their poor generalization ability in response to load variations. A comparison of the performances of the MC-CNN and DWMFCNN models in Tasks T1, T2, and T3 reveals that the dynamic weighting module for multi-scale features in the DWMFCNN model enhances the model’s sensitivity to fault features while reducing its sensitivity to load variations.

**Figure 11:**
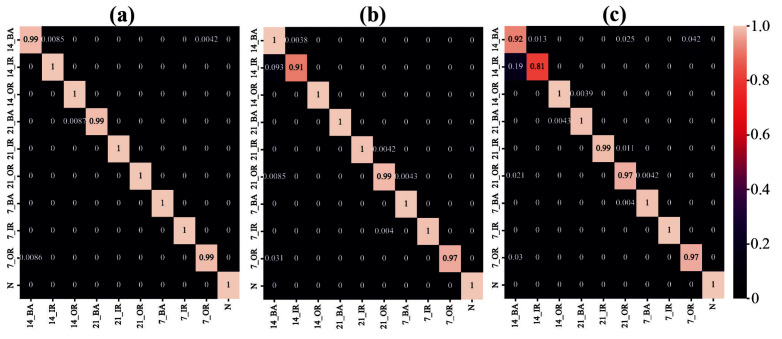
Confusion matrix of three models in Task T1: (**a**) DWMFCNN, (**b**) MC-CNN, and (**c**) CNN.

To analyze the classification accuracy of the three models for each fault type more precisely, the confusion matrix for each model in Task T1 is presented in Figure 11. As indicated in Figure 11a, the proposed DWMFCNN model achieves 100% classification accuracy for seven fault types and 99% accuracy for the remaining three. A comparison of the confusion matrices reveals that the DWMFCNN model effectively learns rich and complementary fault features. With the dynamic weighting modulation mechanism, this model excels in tasks involving variable load conditions, accurately classifying fault data across different loads.

**Figure 12:**
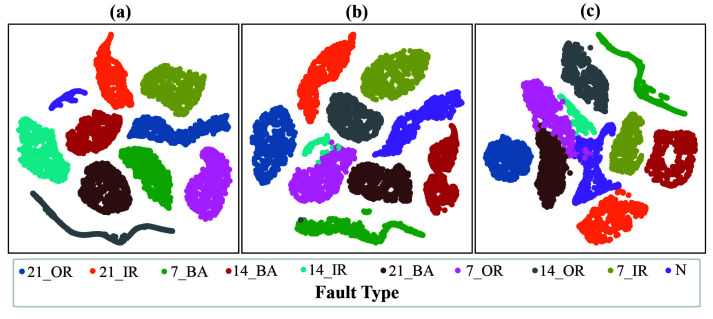
Feature visualization of three models in Task T1: (**a**) DWMFCNN, (**b**) MC-CNN, and (**c**) CNN.

To intuitively assess and better understand the classification performance of three models in Task T1, features output from one layer of each model after the Flatten layer are projected into two dimensions for visualization using t-SNE. The results are displayed in Figure 12. A comparison of Figure 12a–c reveals that the proposed DWMFCNN model effectively learns the features of different fault types, fully separating them and demonstrating the strongest fault discrimination capability among the three models. In contrast, the other two comparative models exhibit weaker fault feature learning abilities. The MC-CNN model achieves near separation of eight fault types but fails to fully distinguish between two specific fault types: 14_IR and 7_OR. Meanwhile, the CNN model shows that four fault types remain mixed even after feature learning.

#### 4.3.2. The Effectiveness of the Proposed Method for Fault Diagnosis Under Variable Speed Conditions

**Figure 13:**
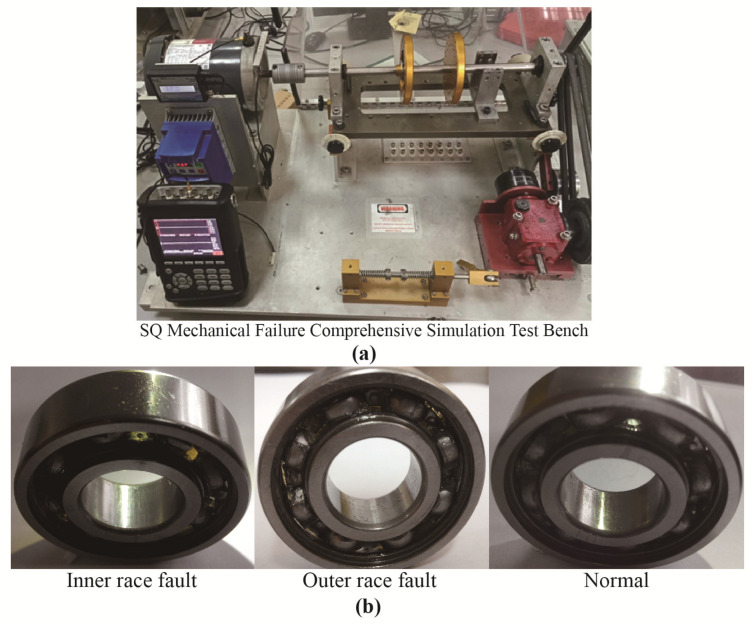
(**a**) Experimental setup for bearing-fault diagnosis. (**b**) Demonstrations of bearing failures.

To validate the fault diagnosis performance of the proposed method in real variable speed industrial scenarios, this section utilizes a dataset obtained from a bearing test bed with speed variations over time. The experiment employs the Mechanical Failure Comprehensive Simulation Test Bed from SQ (Spectra Quest) Company to simulate faults in both the outer and inner rings of motor bearings, as illustrated in Figure 13a. The test setup consists of three main elements: the motor, the rotor, and the load. It employs piezoelectric acceleration sensors to capture signals from the motor bearings at a sampling rate of 25.6 kHz. The bearing model used is NSK6203, with the defective bearing located at the drive end of the motor. The dataset is divided into three health conditions for the bearings: inner race fault, outer race fault, and normal condition. Each of these health states is further segmented into three levels of fault severity: D1 (mild), D2 (moderate), and D3 (severe). The three bearing health states corresponding to D1 (mild) are depicted in Figure 13b, from left to right.

**Figure 14:**
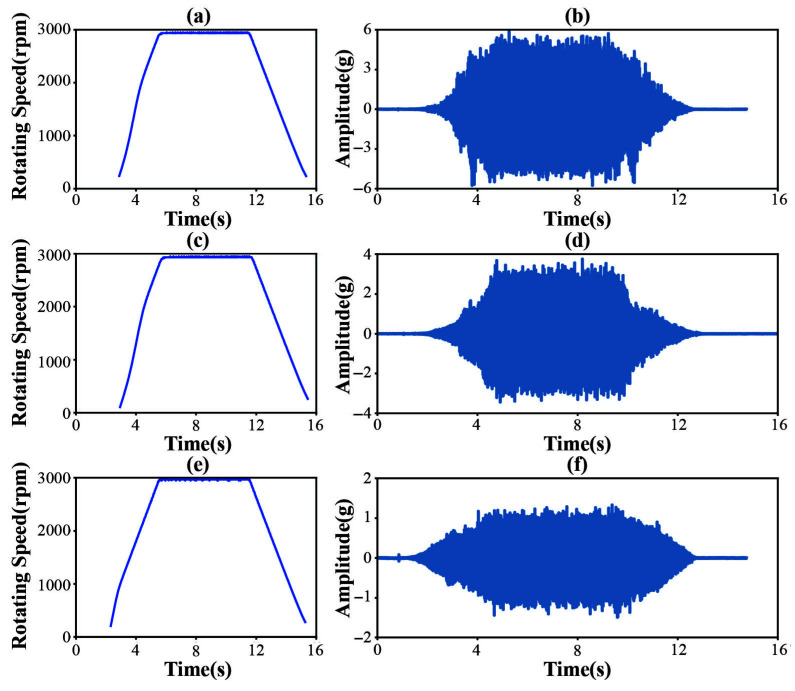
Vibration signal waveforms and their corresponding rotational speed change curves for the three bearing health states in D1. (**a**,**b**) Inner race fault. (**c**,**d**) Outer race fault. (**e**,**f**) Normal.

Each experiment adhered to the protocol outlined in Table 5, capturing the entire start/stop cycle (0–3000 rpm) during each 15 s acquisition to evaluate the diagnostic effectiveness of the proposed method when subjected to continuous rotational speed variations. This approach is deemed more demanding and realistic compared to a fixed-speed test. Figure 14 shows the vibration signal waveforms of the three bearing health states in D1 (inner race fault, outer race fault, and normal) along with their corresponding speed change curves, presented sequentially from top to bottom.

Table 6 presents four fault diagnosis methods suitable for variable operating conditions, selected to evaluate the performance of the proposed methods in this paper for fault diagnosis under variable speed conditions. All experiments are conducted within the Keras framework to assess each method’s performance across D1, D2, and D3. The results are averaged over ten trials to mitigate the influence of random factors on model performance. Methods M1-M4 all use the original vibration signals as inputs, and in method M5 (Proposed method), the original vibration signals are used as inputs to the DWMFCNN model after multiscale denoising based on the AD test. During the testing process, the training epoch of the five methods is set to 100, the batch size is set to 64, the optimization algorithm uses Adam with a learning rate of 1 × 10^−3^, and the other four comparative models are set with appropriate hyperparameters in accordance with the corresponding literature to obtain better performance to further validate the methods proposed in this paper.

**Figure 15:**
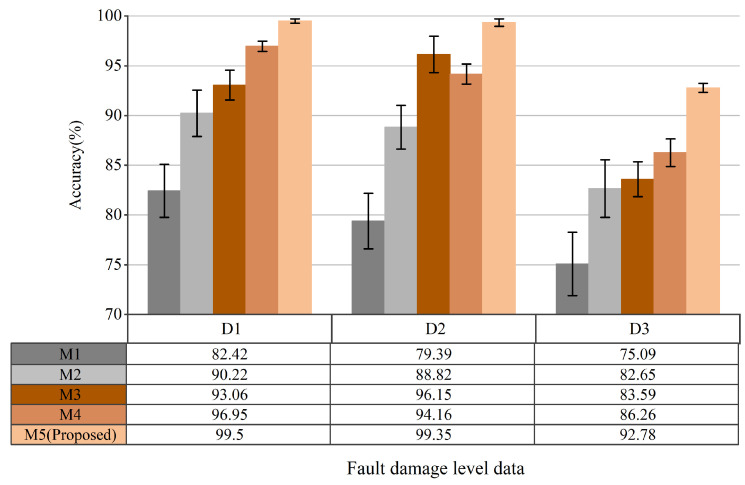
Performance comparisons of five models in three fault damage levels data (D1–D3).

Figure 15 shows the accuracy levels obtained by the five compared methods in the three fault damage level data D1, D2, and D3 variable speed condition tasks. Three conclusions can be drawn from Figure 15: (1) The dual multiscale mechanism for noise reduction and feature extraction in method M5 yields the highest accuracy among the five algorithms, indicating its potential for industrial applications involving unknown noise distributions and variable RPM issues. (2) The performance of methods M1-M4 differs significantly from that of method M5 proposed in this paper on the variable speed dataset. Methods M1 and M2 focus solely on denoising vibration signals and achieving classification accuracy, neglecting the model’s domain adaptation under variable operating conditions, which results in a lower accuracy. In contrast, method M5 can extract fault-sensitive features unaffected by operating conditions and enhance the model’s adaptability to changes in rotational speed and load. Method M3 improves the model’s domain generalization under variable conditions but fails to address noise effects on performance. Although method M4 enhances domain adaptability, it only employs a wider convolution kernel to suppress high-frequency noise that does not affect the original vibration signals. The high-frequency noise does not consider the noise distribution in the original vibration signal, resulting in a poor model performance in practical applications with unknown noise distributions. Conversely, method M5 estimates the noise distribution in the original vibration signal and performs multi-scale denoising, demonstrating robustness in noise and mode mixing.

**Figure 16:**
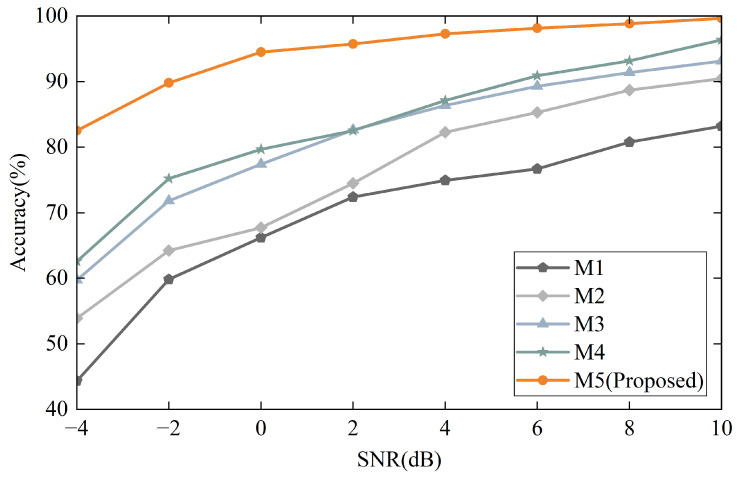
Performance comparisons of five models in noisy environments with different SNRs.

Eight levels of Gaussian white noise (−4, −2, 0, 2, 4, 6, 8, 10) were added to the D1 dataset to create the noise signal. Figure 16 presents the diagnostic outcomes of the proposed method alongside four comparative methods under variable speed conditions at various SNR levels. The results indicate that the diagnostic accuracy of all five methods declines as the SNR decreases, demonstrating that their performance is influenced by ambient noise, which interferes with and obscures the information within the vibration signals. Among the five methods, the proposed method exhibits the highest performance across varying environmental noise levels, suggesting that the multi-scale noise reduction mechanism in the proposed dual multi-scale framework significantly enhances the model’s noise resilience. A comparison between method M2 and the proposed method M5 reveals that the multiscale feature learning mechanism in M5 extracts more robust features from noisy input signals compared to the single-scale CNN. These findings indicate that the proposed method is suitable for fault diagnosis under variable operating conditions in real-world noisy industrial environments.

## 5. Conclusions

This paper presents a method for diagnosing faults in rolling bearings (VMD-AD+DWMFCNN) that utilizes a dual multiscale mechanism suitable for operating conditions characterized by noise and variability. This method includes a multi-scale denoising method along with multi-scale feature extraction and dynamic weighting approach. The primary contributions are summarized as follows:

(1) The multi-scale denoising method eliminates the need to predefine a noise distribution model during noise detection. Instead, it employs a data-driven approach to accurately fit the noise distribution within the signal. The noise detection based on the AD statistical distance effectively suppresses noise, yielding a denoised reconstructed signal that closely resembles the original.

(2) The method utilizes multi-scale intrinsic mode functions (IMFs) obtained through VMD decomposition to perform noise detection and removal across each component, effectively separating noise from the mixed multi-scale mode signals.

(3) Leveraging the DWMFCNN model, this method extracts comprehensive and condition-independent fault-sensitive features that enhance the adaptability of the system to variations in noise, speed, and load during operation.

(4) This method demonstrates a strong capability for fault feature extraction and achieves high accuracy in fault-type identification across varying operating conditions.

In our upcoming research, we intend to refine the suggested model for the complete adaptive extraction of features from the initial vibration signals. Our emphasis will be on boosting the model’s ability to generalize and ease domain transfer. Furthermore, we will investigate innovative approaches to enhance the performance of the model, with the ultimate goal of improving the precision in diagnosing faults in rolling bearings.

## Figures and Tables

**Table 1:** The details of CWRU bearing dataset.

Fault Types Description	Samples				Category
	**0 hp**	**1 hp**	**2 hp**	**3 hp**	
N (normal)	300	300	300	300	Class 1
7-OR (outer race fault diameter: 0.007 inch)	300	300	300	300	Class 2
14-OR (outer race fault diameter: 0.014 inch)	300	300	300	300	Class 3
21-OR (outer race fault diameter: 0.021 inch)	300	300	300	300	Class 4
7-BA (ball fault diameter: 0.007 inch)	300	300	300	300	Class 5
14-BA (ball fault diameter: 0.014 inch)	300	300	300	300	Class 6
21-BA (ball fault diameter: 0.021 inch)	300	300	300	300	Class 7
7-IR (inner race fault diameter: 0.007 inch)	300	300	300	300	Class 8
14-IR (inner race fault diameter: 0.014 inch)	300	300	300	300	Class 9
21-IR (inner race fault diameter: 0.021 inch)	300	300	300	300	Class 10

**Table 2:** Evaluation indicators of denoising methods.

Denoising Methods	SNR Gain (dB)	RMSE
VMD	1.886	0.503
VMD-AD	5.147	0.162

**Table 3:** Fault-diagnosis-task-specific settings for CWRU bearing dataset under different operating conditions.

Task	T1	T2	T3
Train	0 hp + 2 hp + 3 hp	0 hp + 1 hp + 3 hp	0 hp + 1 hp + 2 hp
Test	1 hp	2 hp	3 hp

**Table 4:** The details of the architecture of DWMFCNN.

Module Name	Layer Name	Convolution Kernel /Pooling Size	Number of Convolution Kernels	Activation Function
Multiscale Feature Extraction Module	Conv2d	(16,16)	4	ReLU
	Pooling	(4,4)	/	
	Conv2d	(6,6)	4	
	Conv2d	(4,4)	4	
	Conv2d	(2,2)	4	
	Concatenate	/	/	
Multiscale Feature Dynamic Weighting Module	Conv2d	(4,4)	4	
	Global average pooling	(2,2)	/	
	FC layer	5	/	
	FC layer	10	/	
	Weighting layer	/	/	
Multiscale Feature Fusion Module	Conv2d	(4,4)	4	
	Batch Normalization	/	/	
	Pooling	(2,2)	/	
	Flatten	/	/	
Fault Classification Module	FC layer	100	/	
	Batch Normalization	/	/	
	FC layer	10	/	Softmax

**Table 5:** Parameters of continuous speed change experiments.

Category	Parameter	Value/Description	Remarks
Data Acquisition	Sampling frequency	25.6 kHz	Anti-aliasing filtered
	Duration per experiment	15 s	0→3000→0 rpm cycle
	Health conditions	N, IF, OF	N: Normal; IF: Inner race fault; OF: Outer race fault
Speed Profile	Acceleration rate	200 rpm/s	Linear ramp
	Maximum speed	3000 rpm	5 s dwell at maximum

**Table 6:** Five methods of comparison.

Method	Description
M1	WGWOA-VMD-SVM [15], a rolling-bearing-fault diagnosis method based on WGWOA-VMD-SVM.
M2	VMD+CNN [44], a novel feature extraction and fault diagnosis method for planetary gears based on VMD, SVD, and CNN.
M3	CNN-C [16], a fault diagnosis method for unknown working conditions.
M4	WDCNN [20], a fault diagnosis method with noise resistance and domain Adaptation Capability.
M5	VMD-AD+DWMFCNN (proposed method), a rolling-bearing-fault diagnosis method based on a dual multi-scale mechanism applicable to noise-variable operating conditions proposed in this paper.

## Data Availability

The raw data supporting the conclusions of this article will be made available by the authors on request.
